# C-Reactive Protein and Cancer—Diagnostic and Therapeutic Insights

**DOI:** 10.3389/fimmu.2020.595835

**Published:** 2020-11-19

**Authors:** Peter C. Hart, Ibraheem M. Rajab, May Alebraheem, Lawrence A. Potempa

**Affiliations:** Roosevelt University, College of Science, Health and Pharmacy, Schaumburg, IL, United States

**Keywords:** C-reactive protein, monomeric C-reactive protein, inflammation, tumor microenvironment, acute phase response

## Abstract

Cancer disease describes any pathology involving uncontrolled cell growth. As cells duplicate, they can remain localized in defined tissues, forming tumor masses and altering their microenvironmental niche, or they can disseminate throughout the body in a metastatic process affecting multiple tissues and organs. As tumors grow and metastasize, they affect normal tissue integrity and homeostasis which signals the body to trigger the acute phase inflammatory response. C-reactive protein (CRP) is a predominant protein of the acute phase response; its blood levels have long been used as a minimally invasive index of any ongoing inflammatory response, including that occurring in cancer. Its diagnostic significance in assessing disease progression or remission, however, remains undefined. By considering the recent understanding that CRP exists in multiple isoforms with distinct biological activities, a unified model is advanced that describes the relevance of CRP as a mediator of host defense responses in cancer. CRP in its monomeric, modified isoform (mCRP) modulates inflammatory responses by inserting into activated cell membranes and stimulating platelet and leukocyte responses associated with acute phase responses to tumor growth. It also binds components of the extracellular matrix in involved tissues. Conversely, CRP in its pentameric isoform (pCRP), which is the form quantified in diagnostic measurements of CRP, is notably less bioactive with weak anti-inflammatory bioactivity. Its accumulation in blood is associated with a continuous, low-level inflammatory response and is indicative of unresolved and advancing disease, as occurs in cancer. Herein, a novel interpretation of the diagnostic utility of CRP is presented accounting for the unique properties of the CRP isoforms in the context of the developing pro-metastatic tumor microenvironment.

## Introduction

Cancer is a pervasive disease affecting many people across the globe. Worldwide, the prevalence of people living within 5 years of receiving a cancer diagnosis is estimated to be 43.8 million. In 2018, 18.1 million new cancer cases were diagnosed worldwide, and 9.6 million deaths were reported. The most reported new cases involve the lung and breast, with colorectal cancer third, prostate cancer fourth, and stomach cancer fifth (1, 2). Each reported cancer is associated with variable overall life expectancies, years of life lost, and 5-year survival rate (3–6).

The term cancer describes a physiological condition in which body cells grow and replicate in an uncontrolled and unregulated fashion. While cancer is often categorized by the tissue in which the predominant uncontrolled growth originates, the disease is fundamentally defined by a loss of basic cellular processes that regulate proliferation. When cells proliferate, their mass and the mass of ancillary tissues in which the growth occurs increases, leading to localized areas of disrupted tissues which can overwhelm the natural protective immune responses that are designed to identify and destroy aberrantly growing cells, and to repair damaged tissues. Hence, in evaluating cancerous disease, consideration must be given not only aberrant cells, but the tissues, vasculature, and immune responses in which the malignant growth occurs. If cancerous growth is relatively slow and localized, treatment options include surgical resection and radiotherapy. If cancerous growths become unregulated and rapid, involved tissues are compromised and weakened, and tumor cells can break free to metastasize to other tissues. Treatments options for disseminated cancers become limited and prognosis for long term survival decreases. Strategies for treating cancers must therefore include an assessment of how tissue structures involved in cancerous growths may be compromised and how natural barriers might be strengthened to counteract the growth of tumor masses and to better coordinate the immune responses against tumor development.

Both innate and adaptive immune responses exist to recognize and remove diseased cells, primarily involving innate system humoral factors and natural killer cells. Innate responses involve neutrophils, M1 macrophages, natural killer cells, interferons, cytokines, and the acute phase response (7). Adaptive responses involve antibodies developed to specifically recognize malignant cells, and direct cytotoxic T-lymphocytes to specifically recognize intracellular abnormalities associated with uncontrolled cellular duplication that often display tumor specific antigens, leading to cell-mediated tumor cell apoptosis (8). However, a number of cancers present with “cold” tumors, showing limited or no infiltration of activated T cells within the tumor compartment (9). Many cancers are known to produce suppression factors that target effective immune system surveillance, thus giving cancer cells an advantage in the balance between continued proliferation and leukocyte-mediated apoptosis. The exciting development of biotherapeutic reagents known as checkpoint inhibitors, which function by neutralizing the immunosuppressive factors [i.e. by directly binding to Program Death Ligand-1 (PDL-1) or its receptor (PD-1)], shifts the balance back to natural immune function, slowing or stopping tumor cell growth in treated individuals (10). This is likely due to the prevention of PD-1-dependent T cell exhaustion; therefore, blockade increases the presence of cytotoxic T cells in the microenvironment capable of tumor cell lysis through perforin and granenzyme secretions (11). While promoting the cytotoxic capacity of the local immune response can slow or prevent tumor growth in some cases (12), it is notable that certain distributions of leukocytes can also favor a state of chronic inflammation that conversely would promote genomic instability, survival, and proliferation of tumor cells that can confer tumor development and progression [reviewed in (13)]. Thus, the dualistic nature of the immune system in cancer suggests that tight regulation of immune and inflammatory responses is necessary to prevent tumor growth and metastasis.

This report focuses on innate immunity and the acute phase response (APR) to cancerous growth. More specifically, it provides a new perspective on the diagnostic and therapeutic value of the prototypic acute phase reactant, C-reactive protein (CRP), and cancer. The concepts presented herein represent a unifying understanding of the value of CRP as a key protein of the natural inflammatory response and how both beneficial and pathological inflammation contribute to cancerous disease as well as the pathologies that involve any tissue damaged by trauma or disease.

## Use of CRP as a Diagnostic Marker in Cancer

The US Food and Drug Administration (FDA) guidance for assessing the relevance of CRP as a diagnostic marker in any disease involving a host defense inflammatory response, describes CRP as a single protein that can be reliably measured from blood using various qualitative, semi-quantitative and quantitative measurements (14). Two concentration thresholds were established: 1.) conventional CRP levels (defined as CRP levels ≥ 10 µg/ml), and 2.) high sensitivity CRP levels (i.e. hsCRP; defined as CRP levels < 10 µg/ml). FDA guidance does not associate either CRP or hsCRP levels with specific diseases or risks for disease, cautioning that any interpretation of CRP levels must include the context of a specific clinical evaluation. However, high levels of CRP were found to be strongly associated with advanced disease severity in numerous cancer types (elaborated below). Hence, CRP measurements have potential utility as a diagnostic tool in assessing disease status and progression, including in cancer.

**Figure 1:**
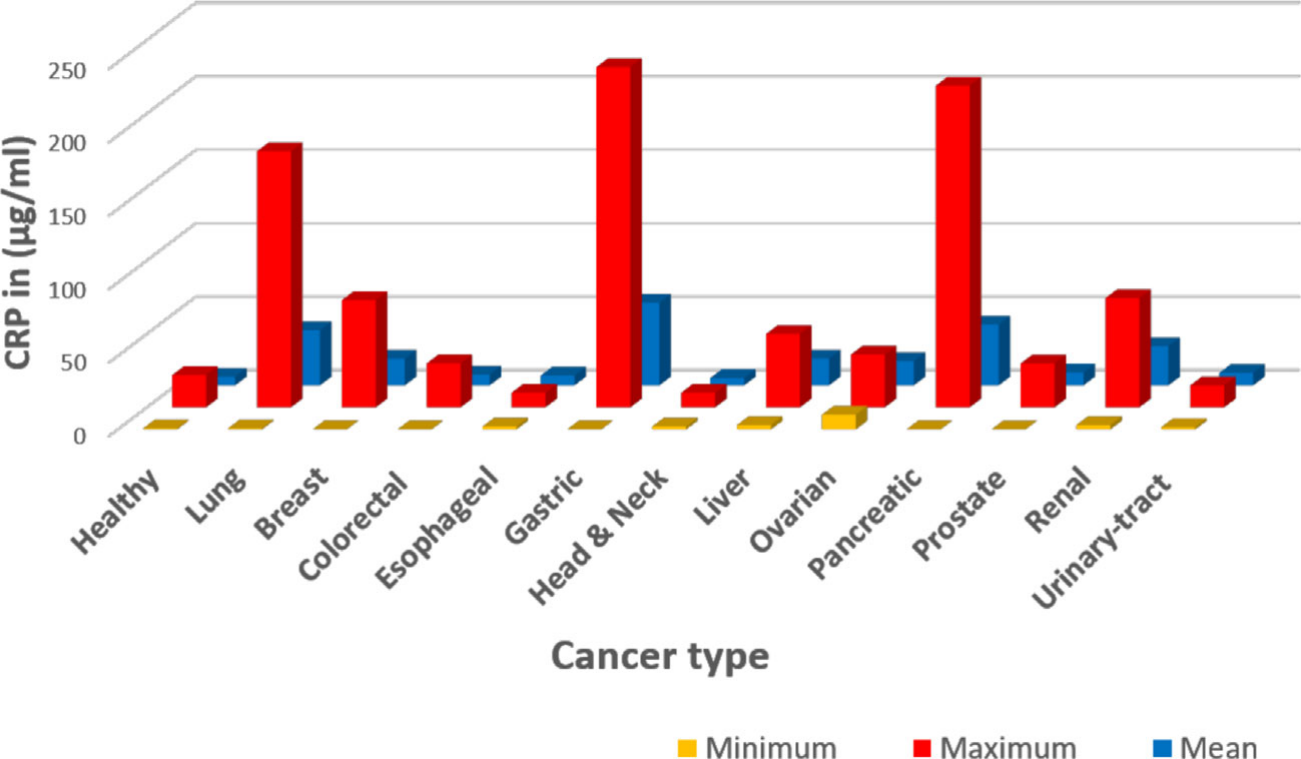
Graphic representation of data summarized in **Table 1**. The CRP values were extracted from published references as detailed in **Table 1**. In this presentation, data were tabulated in Microsoft Excel based on cancer type, then the Excel functions were used in calculations: Minimum describes the lowest reported level; Maximum describes the highest reported level; and Mean describes the average of the reported values. One limitation of the reported data summarized here is the lack of specific clinical conditions ongoing when (or how frequent) CRP values were collected and measured.

A comprehensive review of the literature on the diagnostic significance and therapeutic value of CRP blood levels in cancer proved to be problematic. Reported CRP levels varied from <1 μg/ml to more than 175 μg/ml [(15); see **Table 1** and **Figure 1**] and were most often reported with reference to the tissues affected with cancerous growths (e.g. lung, breast, gastrointestinal, esophageal, head and neck, sexual and reproductive organs, renal, pancreas, and blood). One complication to interpreting the value of CRP measurements in any such disease condition is that its relative blood level can change rapidly and in direct relationship to the stage and extent of progressive disease and/or associated complication (e.g. infections) that may accompany the disease progression. Another limitation is that many of these studies did not fully characterize the relationship between CRP and the discrete variables that define cancer staging (e.g., tumor size in TNM staging of breast cancer, etc.); however, the associations drawn between CRP with prognosis and disease severity are consistent among cancers and indicate the potential utility of CRP as a prognostic index. Reports of CRP level in relationship to blood albumin level (i.e. the CRP/albumin ratio) as a novel prognostic index for survival for cervical cancer have appeared (64). In addition, the prognostic value of CRP in relationship to the neutrophil/lymphocyte ratio has also been proposed. Since CRP levels change rapidly during disease, some studies describe a “maximal CRP level” as a relevant diagnostic index. However, such values must be evaluated with reference to how prolonged cancer disease extends, the treatments used during disease, and the levels measured both before and after any surgical intervention. Nonetheless, these maximal CRP values have been discussed as one criterion for assessing the extent of disease and the prognosis for recovery (27, 102).

**Table 1:** Overview of CRP values reported in various cancers distinguishing Conventional CRP levels (≥ 10 μg/ml) from High Sensitivity (hsCRP) levels (< 10 μg/ml).

Tissue affected by cancer	Notes and generalized conclusions	CRP levels reported*		References**
		Conventional CRP(≥ 10 μg/ml)	hsCRP(1-10 μg/ml)	
**Lung** **Non-Small Cell**	• Higher conventional levels correlate with tumor size and staging (2) • Higher levels (before or after surgery) are indicators of poor prognosis (1) • Smoking and hsCRP levels did not correlate with increased risk for lung adenocarcinoma (9) • Smoking and lung function with hsCRP can predict bronchial dysplasia than can progress to cancer (14) • Conventional CRP above 40 μg/ml is predictive of metastasis (17) • CRP levels do correlate with level of inflammation which is more pronounced in cancer than COPD (18)	4–20 (1) >20 (1) 13.4 ± 8.6 (2) 114.2 ± 60.1 (2) 84 ± 88 (5) ≥10 (7) 7.1–100 (8) ≥10 (9) 1–30 (10) 4.14–87.9 (11) 143 max (12) >101–122.6 (15) >40 (17) 22.49 ± 2.31 (18) 20.42 ± 1.95 (18) 10.27 + 2.12 (19) 66.35 (20) 71.20 (20) 96.30 (20)	<3 (1) 5.4 ± 9 (3) 6.4 ± 7.9 (3) <1 (4) ≥5.6 (4) <5 (5,6, 16) ≥5 (5,6) 0–0.7 (11) 0.7–1.76 (11) 1.77–4.13 (11) 3 (12) 1–5 (13) >0.5 (14) 8.37 + 0.91 (18)	(1) Alifano et al. (16) (2) Aref and Refaat (17) (3) Bittoni et al. (18) (4) Chaturvedi et al. (19) (5) Hara et al. (20) (6) Hara et al. (21) (7) Jin et al. (22) (8) Jing et al. (23) (9) Koch et al. (24) (10) Liao et al. (25) (11) Muller et al. (26) (12) Pastorino et al. (27) (13) Shinohara et al. (28) (14) Sin et al. (29) (15) Szturmowicz et al. (30) (16) Tomita et al. (31) (17) Torrecilla et al. (32) (18) Vagulienė et al. (33) (19) Wei et al. (34) (20) Zhao et al. (35)
**Breast**	• Elevated conventional CRP levels are associated with reduced overall and disease-free survival and increased risk of death (21) • hsCRP levels are not predictive in post-menopausal breast cancer incidence or in apparently healthy women (25)	≥16.4 (21) 12 ± 8 (22) 42 ± 12 (22) >10 (23,24) 0.1–39.5 (26) 0.1–73 (26) 0.6–33.6 (29) >10 (30)	1.5–10 (24,25) 4.93 ± 6.65 (26) 5.26 ± 8.59 (26) 1.0 ± 1.3 (27) 1.5 ± 1.7 (27) >5 (28) 5.1 + 5.3 (29) >3 (31) 2.6 (32)	(21) Allin et al. (36) (22) Asegaonkar et al. (37) (23) Gathirua-Mwangi et al. (38) (24) Guo et al. (39) (25) Nelson et al. (40) (26) Rodriguez-Gil et al. (41) (27) Sabiston et al. (42) (28) Sicking et al. (43) (29) Thomson et al. (44) (30) Villaseñor et al. (45) (31) Wang et al. (46)(32) Zhang et al. (47)
**Colon and Rectum**	• Higher hsCRP associates with higher risk for colon but not rectal cancer (33) • Conventional CRP levels associate with colorectal cancer mortality (36) • CRP gene rs1205 polymorphism was not associated with the risk of colorectal cancer (35)	>10 (36,38) 30 (37) 0–196 (37) 0.01–22.8 (41)	1.1–5.6 (33) 1–4.7 (33) 2.69 (34) 1.97 (34) <2.1 (36) 2.2–5.0 (36) 5.1–10 (36) 3 (39) 4.1 ± 3.2 (39) 1–10 (39,42,43) 1.07–4.36 (40) 2.86–5.20 (40) 0.6 (41)	(33) Aleksandrova et al. (48) (34) Erlinger et al. (49) (35) Fang and Ye (50) (36) Goyal et al. (51) (37) Holm et al. (52) (38) Ishizuka et al. (53) (39) Lumachi et al. (54) (40) Nimptsch et al. (55) (41) Shibutani et al. (56) (42) Toiyama et al. (57) (43) Zhou et al. (58)
**Esophagus**	• Elevated serum CRP was associated with poor overall survival (44-47)		≤10 (44) 5–10 (45) 3 (46) 2–10 (47)	(44) Badakhshi et al. (59) (45) Huang et al. (60) (46) Katano et al. (61) (47) Zheng et al. (62)
**Gastrointestinal Tract**	• Elevated conventional CRP is associated with progressive disease and advanced stage disease and correlates with worse survival (49) • Higher conventional CRP levels correlate with advanced stage metastatic cancer (50) • Post-operative levels are useful to monitor infections (51)	17 (48) 0.08–231.7 (49) ≥10 (50,52) 177 (51)	<3 (49) 1.6 (49)	(48) Baba et al. (63) (49) Chang et al. (64) (50) Shimura et al. (65) (51) Shishido et al. (66) (52) Yu et al. (67)
**Head and Neck**	• Pre-operative levels are prognostic of oral cancer (55) • Elevated levels associate with worse prognosis (53) • CRP may contribute to tongue squamous cell carcinoma (56) • CRP levels associate with perceived pain and inflammatory process (57)	<20 (57)	2–8 (53) <10 (54) ≥5 (55)	(53) Fang et al. (68) (54) He et al. (69) (55) Tai et al. (70) (56) Du et al. (71) (57) Oliveira et al. (72)
**Liver**	• CRP levels correlate with hepatocellular carcinoma aggressiveness (58) • Elevated CRP correlates with poor prognosis and is useful in staging (59)	10–50 (58) >50 (58) ≥ 10 (59)	<10 (58)	(58) Carr et al. (73) (59) Kinoshita et al. (74)
**Reproductive Organs** • **Ovary** • **Prostate**	• Elevated CRP associates with increased risk and survival • Higher Levels are measured in platinum-resistant ovarian tumors (68) • CRP/Albumin ratios are prognostic (69) • Pre-diagnostic hsCRP levels are not predictive of risk (65) • CRP levels are not as predictive in clinically localized prostate cancer compared to advanced disease (66) • High levels associate with metastasis and worse survival (64)	36 ± 48 (60) 60 ± 66 (60) 28 ± 38 (60) >10 (61; 62) <12 (63) 0.02–29.9 (64)	≤10 (61,68,69) 1.24 ± 2.94 (65) <4 (66) ≥8.6 (67)	(60) Hefler et al. (75) (61) Li et al. (76) (62) Lundin et al. (77) (63) Graff et al. (78) (64) Liu et al. (79) (65) Platz et al. (80) (66) Schnoeller et al. (81) (67) Thurner et al. (82) (68) Xu et al. (83) (69) Liu et al. (84)
**Pancreas**	• Elevated levels associate with poor outcomes (70) • Neutrophil-lymphocyte ratios with CRP levels have prognostic value (71)	3–50 (70) 0.1–219 (71)	>4.5 (71) 3–10 (72)	(70) Chen et al. (85) (71) Inoue et al. (86) (72) Stevens et al. (87)
**Kidney and Urinary tract**	• CRP levels can predict mortality (73), treatment outcome and tumor recurrence in solid tumor renal cell carcinoma and digestive tumors [Shotriya et al. (15)] • Preoperative values are predictive of survival and end stage disease requiring hemodialysis (74) • Elevated preoperative levels correlate with aggressive tumor biology (76) • Elevated CRP/Alb ratio correlate with poor survival after or partial nephrectomy (78)	24.3 ± 50.1 (73) 1.5–15 (77)	<5 (74) <3 (75) <9 (76)	(73) Hsiao et al. (88) (74) Omae et al. (89) (75) Teishima et al. (90) (76) Aziz et al. (91) (77) Dai et al. (92) (78) Guo et al. (93)
**Blood**	• Higher CRP levels are a poor prognostic indicator in large B-cell lymphoma (79) • Measuring CRP has value in melanoma (80)	<15 (79)	<10 (80)	(79) Troppan et al. (94) (80) Fang et al. (95)
**Sarcomas**	• Increased preoperative CRP is prognostic of poorer outcomes in bone cancer (82) and Soft tissue sarcoma (84)	43 (85) 0.1–342 (84) ≥8 (81, 82) 0.1–34.2 (83)		(81) Fang et al. (96) (82) Li et al. (97) (83) Nakamura et al. (98); Nakamura et al. (99) (84) Wang et al. (100) (85) Yanagisawa et al. (101)
**Adult Solid Tumors: Renal Cell Solid Carcinoma & Digestive Solid Tumors**	• CRP as a predictor of prognosis, treatment outcome or tumor recurrence (86)	>10, >35, >50, >150 (86)	<1 – >9.8 (86)	(86) Shrotriya et al. (15)

*Conventional CRP levels (≥ 10µg/ml) are summarized separately from high sensitivity levels (hsCRP: < 10µg/ml) to help differentiate FDA defined diagnostic relevance from putative microinflammatory responses suggested by reported levels of hsCRP.

**The parenthetical number assigned to each reference is used to indicate where the CRP and hsCRP values, notes and general conclusions are derived within this table.

Another complicating factor in interpreting the diagnostic and therapeutic value of CRP in cancer disease is the current focus on the value and significance of “high sensitivity CRP” levels (i.e. hsCRP). Baseline levels of CRP in purportedly healthy individuals are generally described as being <1–2 μg/ml. Some studies reviewed and included in reports summarized in **Table 1** describe and differently interpret cohort groups with hsCRP levels between 1–3 and >3 μg/ml. While FDA offers no guidance on the diagnostic significance of such values in any disease, including cancer, it is important to note that numerous reports have appeared that associate baseline hsCRP levels more generally with populations of individuals grouped by gender, age, ethnicity, degree of fitness, and obesity. Indeed, with sensitive assays for CRP measurement now readily available, including point-of-care measurements where CRP values can be generated from a finger stick drop of blood in minutes, thousands of reports have appeared reporting that slightly elevated hsCRP levels are reflective of increased risk for developing and exacerbating cancerous growth (see **Table 1** for a list of references that discuss hsCRP in the defined cancers).

**Table 1** offers one literature overview summarizing reported CRP blood levels as a function of cancer type, conventional CRP levels reported (i.e. CRP levels ≥ 10 μg/ml), high sensitivity CRP levels reported [hsCRP levels reported (< 10 μg/ml)] and stage of cancer disease and/or complications. A graphic presentation of reported CRP maximum, minimum and mean (average) values as a function of cancer type is presented in **Figure 1**.** **A simplified interpretation of all the data included in **Table 1** is presented in **Table 2**.

**Table 2:** Most consistent interpretations of the diagnostic significance of CRP in cancer.

1.	Plasma CRP is not selective for any cancer type or tissue involving cancer.
2.	Elevated CRP levels (> 10 μg/ml) are associated with active, advanced cancer disease.
3.	Elevated CRP levels (> 10 μg/ml) can be diagnostic of complicating pathologies (e.g. infections).
4.	Significantly, elevated CRP levels (above 50–100 μg/ml) are associated with advanced disease, metastasis, and poor response prognosis.
5.	The significance of hsCRP levels in cancer is not yet known and have no proven value.
6.	Higher conventional CRP levels may be predictive of resistance to certain chemotherapeutic treatment (e.g. platinum resistance in ovarian cancer).
7.	Elevated CRP is noted in and associated with aggressive hepatocellular carcinoma.
8.	Any interpretation of the diagnostic significance of CRP requires consideration of when CRP levels are measured in relation to disease activity (i.e. quiescence or rapid growth phase) and in relation to therapeutic treatments and the response to such treatments.

## CRP—A Marker of Tissue Damage First and Inflammation Second

**Figure 2:**
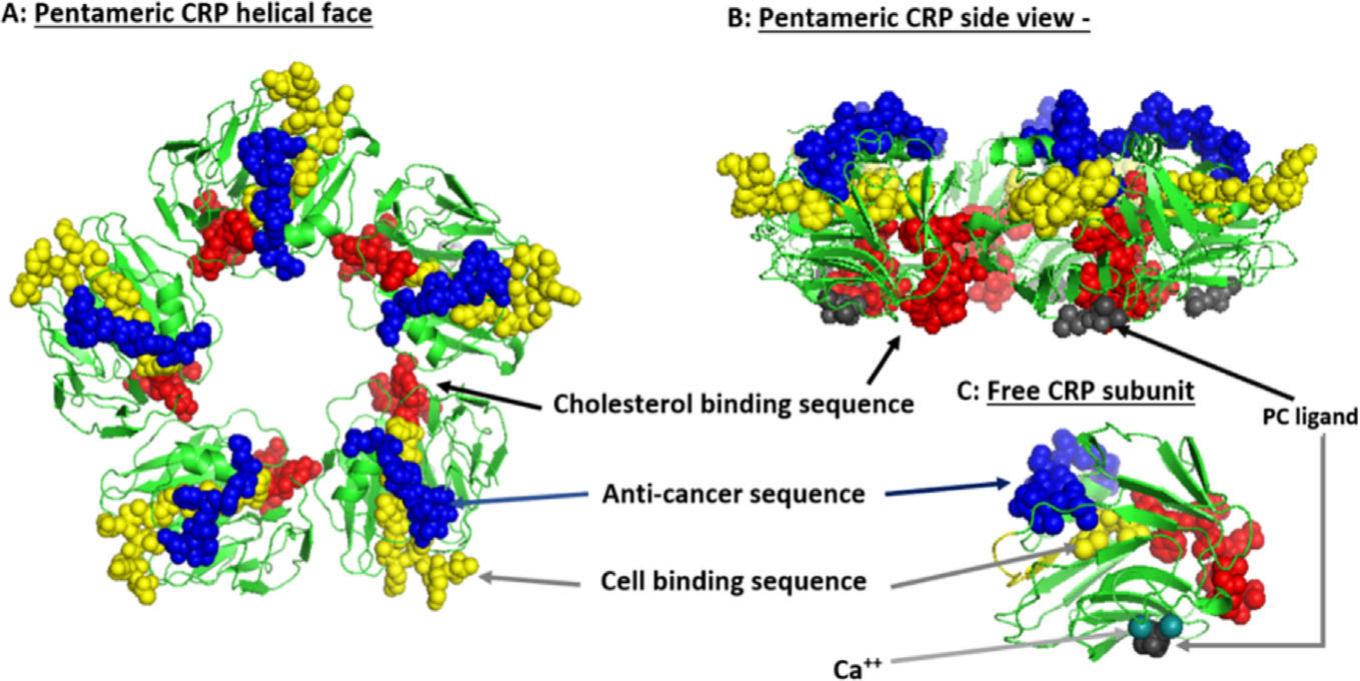
Structural features of serum-soluble pentameric CRP. **(A)** illustrates the location and orientation peptide sequences in CRP reported to have cell-binding activity (shown in yellow and involving _27_TKPLKAFTVCLH_38_) (105), anti-cancer activity (shown in blue and involving _176_LGGPFSPNVL_185_) (106), cholesterol binding activity (shown in red and involving _35_VCLHFYTELSSTR_47_), and which also controls CRP binding to apolipoprotein B, C1q, fibronectin, and collagen (107), in relationship to the phosphocholine (PC) binding face (PC groups shown in gray and involving residues L_64_, F_66_, and T_76_) and bound calcium ions (two per subunit, juxtaposed to each PC binding sites and involves residue E_147_) (PDB code: 1B09; PC and calcium residues as defined by Thompson et al. (108) and Shrive et al. (109), respectively). **(B)** illustrates the orientation of these same residues when the discoid protein is laid flat (i.e. side view). **(C)** shows the orientation of same sequences on the isolated pCRP subunit (note: the exact orientation of these residues on the conformationally changed mCRP subunit has not been determined). The PC ligand binds in a shallow binding pocket controlled by calcium ions, with all PC sites on one face of the flattened discoid structure. The cholesterol binding sequences are near the PC binding sites so that when pCRP binds membrane associated PC, the cholesterol binding sequence is brought into proximity with intra-membraneous cholesterol (in lipid rafts) contributing to the conversion of pCRP into mCRP. The cell binding and anti-cancer peptides are oriented on the opposite face of the discoid protein where they can interact with effector leukocytes and activated inflammatory responses.

Systemic CRP exists as a pentameric structure (pCRP) made up of five subunits, each containing calcium dependent bindings sites that interact with exposed phosphocholine ligand (PC) which can be expressed on activated plasma membrane (103). Exposure of PC groups requires phospholipid remodeling such as occurs with Phospholipase A2 activity or oxidation of acyl chains (104). When pCRP binds to membrane anchored PC, juxtaposed apolar bonding energies contribute to the dissociation of the pentameric isoform into the modified, monomeric isoform (i.e. mCRP) which undergoes structural rearrangement to express a cholesterol binding site as is found in lipid rafts (**Figure 2**) (107, 110).

Since the appearance and concentration of pCRP in blood is not specific for cancer types, tissue locations, or stages of disease, and since its appearance correlates with an ongoing inflammatory response, what is the common denominator that triggers this protein to appear? One reflective focus involves evaluating how CRP may affect the fibrinolytic-like responses that are recognized as hallmarks of cancer growth. Indeed, cancer has been described as “a wound that never heals” (111, 112); any discussion of cancer growth and regulation must, therefore, include an understanding of the structure and function of the extracellular matrix and connective tissues within which the cancers are found. As tumors grow, endothelial cells become activated to allow platelets, neutrophils, and blood proteins (e.g. CRP) to enter tissues as part of normal protective inflammatory response. The goal of this early response is to help control the extent of disease growth and return the tissues to healthy homeostasis. Possible pathways by which CRP may participate in this host defense response include its binding reactivity with 1.) PC ligands which become accessible on stimulated endothelial cell membranes (113, 114), 2.) fibronectin (115–117), 3.) laminin (118), and 4.) collagen (107). CRP is also known to activate and regulate complement activity and bind immune complexes (119). Many reports also detail the interactions of CRP with endothelial cells (119), platelets (120), neutrophils (121), monocytes/macrophages (117, 122), epithelial cells (123), and fibroblasts (124).

**Figure 3:**
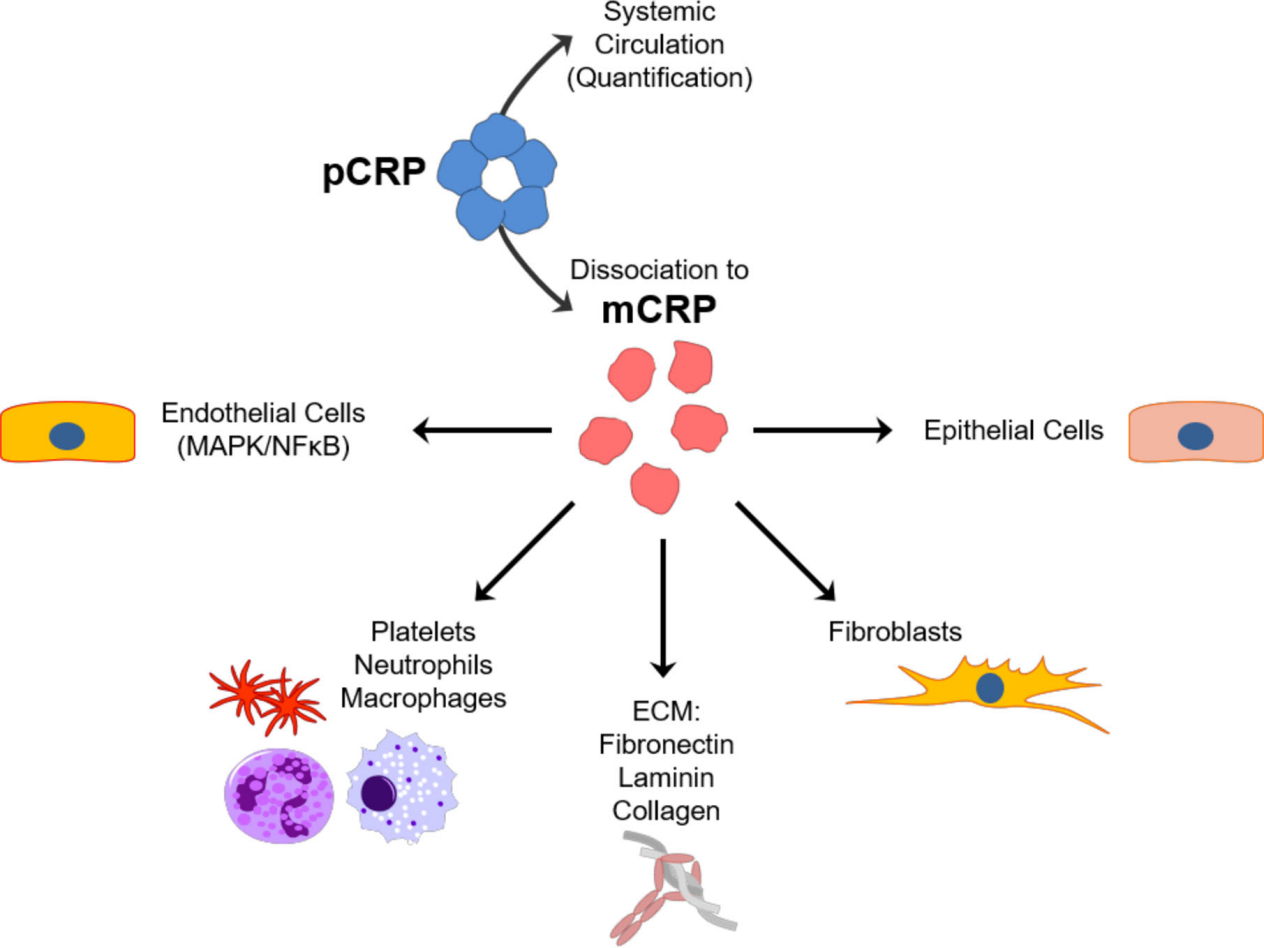
Schematic representation of the predominant interactions of pCRP and mCRP isoforms. Pentameric CRP (pCRP) released from hepatocytes due to inflammation circulates through the systemic vasculature and serves as the pool of quantifiable CRP that is used in diagnostic testing. pCRP, however, once dissociated to monomeric CRP (mCRP) at lipid rafts of cells involved in inflammatory responses instead is highly biologically active. mCRP in turn interacts with a number of different cell types at the sites of inflammation, including endothelial cells, epithelial cells, fibroblasts and immune cells (platelets, neutrophils, macrophages) as well as components of the extracellular matrix (ECM) such as fibronectin, laminin and collagen.

For many decades, the exact role for CRP in the host defense APR remained undefined and controversial as different groups studying similar systems came to opposite conclusions. More recently, as CRP has been shown to exist in more than just a serum soluble cyclic pentamer disc configuration, it is now apparent that the effects of CRP on cell behavior and the tissue microenvironment are clearly dependent on its structural conformation. The highly soluble circulating pentameric CRP (pCRP) binds to PC on the cell surface (e.g., endothelial cells activated by inflammatory signals), which initiates the dissociation of pCRP into its distinct modified, monomeric isoform (mCRP) which has notably reduced aqueous solubility. mCRP will self-aggregate and deposit in tissues or will internalize into plasma membranes and bind cholesterol. Cells activated by mCRP are known to stimulate intracellular signaling pathways, including activating the NFκB transcription factor which propagates intracellular effects known as hallmarks of inflammation (125). Careful comparison studies have now established that mCRP (rather than pCRP) can interact with integral extracellular matrix (ECM) proteins (e.g., fibronectin, collagen) (98). With the awareness and understanding of both the pCRP and mCRP isoforms (including an understanding of how mCRP can be derived from pCRP), and the different roles each has on both cellular and tissue based components involved in acute inflammatory responses occurring in cancer, a consistent role for CRP as a diagnostic marker becomes apparent. A summary of the interactions of CRP with cell types and components of the extracellular environment is summarized in **Figure 3**.

The mCRP isoform can be formed from the pCRP isoform when membranes are activated and cellular responses are stimulated as occurs when tissue are damaged by any means (e.g. trauma, disease, cancerous growth). Once mCRP is formed, it will not reform pCRP; mCRP is readily degraded by proteolytic enzymes and peptides formed by its degradation feedback inhibit many of the acute phase responses stimulated by intact mCRP (126–129). In the earliest minutes of the APR, the rate of conversion of pCRP to mCRP is rapid. Over time, however (hours to days) the rate of conversion of pCRP to mCRP diminishes over time, resulting in quantifiable increases of pCRP in blood [recently reviewed in (130)]. If any injury persists and inflammatory mediators (such as IL-6 and IL-1β) that signal hepatocytes to continue to synthesize acute phase proteins, quantifiable increases in plasma levels of pCRP will result. Increased levels of pCRP, therefore, suggest less pCRP is converted into mCRP. Since mCRP is a potent amplifier of the acute inflammatory response (110, 120, 131–133), any condition that limits its expression will cause a reduction in natural host defense responses. In the case of advancing tumor growth, this would lead to chronic inflammatory signaling that potentiates the “wound that never heals”. As affected tissues are still producing signals that direct the synthesis and release of pCRP, higher blood levels of pCRP are reflective of the persistence and severity of tissue damage associated with cancer growth and progression.

In line with this, CRP levels in blood do indeed correlate with the degree of inflammatory tissue involvement. In soft tissue sarcomas, CRP levels were associated with the degree of tumor infiltration determined by magnetic resonance imaging (MRI) as well as disease-specific survival (99). Similarly, tissue pathologies associated with COVID-19 disease complications identified by computerized tomography (CT) technology were also significantly associated with CRP levels and, importantly, CRP had high sensitivity and specificity to predict severity of the disease (134).

Using this tissue-based perspective as a common denominator, readers are encouraged to interpret CRP diagnostic levels in any clinical situation by first focusing on alterations in tissue structures and second by assessing how it affects or regulates the inflammation that ensues. A brief overview of the extracellular matrix structures and ways that CRP may interact with such structures during inflammatory responses to cancer disease is included below.

## The Extracellular Tissue Microenvironment, Acute Phase Response, and Inflammation

In all animals, including humans, the first line of defense against disease is the structural connective tissue that not only presents a barrier to pathogens and toxins, but also contributes an appropriate macromolecular matrix for coordinated biochemical and immunological host defense reactions. Connective tissues include fibrous proteins, various cells, amorphous ground substance (e.g. proteoglycans and glycosaminoglycans), plasma constituents, ions, and water. Connective tissue can be loose or dense, regular or irregular, fibrous or elastic depending on the types of proteins and polysaccharides that are secreted locally or accumulate in the specific space filled by the connective tissue. The organization and interactions of the components, not only within the framework space but also at boundaries and surfaces, define the physical properties and function of each connective tissue. In tissues as wide ranging as skin and bone, connective tissues form an architectural framework (i.e. the extracellular matrix or ECM) that serves not only as an inert space-filling scaffold, but as a physical structure that plays a dynamic role in organizing and regulating the physiological responses that occur within each tissue. While providing mechanical structure through the deposition of numerous molecules (e.g., collagens, laminins), the ECM also acts as an intermediary space in which growth factors, cytokines, metabolites, and other secreted factors can communicate between cells within tissue compartments (135–137).

When connective tissue is injured either by incision, accident, xenobiotic stress or disease, the APR is activated to stimulate innate host defenses to the injury and the wound healing process so to efficiently and effectively repair the injured tissue (138). These processes must work in concert with hematological and immunological mechanisms that are triggered to defend the body from anything that might threaten the homeostasis affecting not only localized tissues, but of the entire organism as well. The APR reaction involves the initial production of signals locally (e.g., IL-6, IL-1β) at the site of injury, which, once secreted into the extracellular milieu, can have extensive physiological effects on cells within the local tissue or act systemically to promote production of acute phase proteins, such as hepatic CRP (138). This frequent early event during wound repair is characterized by such inflammatory responses that in turn facilitate the rapid recruitment and activation of immune cells, such as neutrophils and other leukocytes. Neutrophils migrate into the wound and scavenge for debris and foreign matter that must be removed. In uncomplicated repair, the movement of neutrophils into a wound is transient and resolved prior to the in-growth of vascular tissue (i.e. granulation tissue). If foreign materials are present, the neutrophil response may persist and thus complicate the wound healing process by having the conflicting processes of removal (scavenging) and repair occurring simultaneously. In such an event, the wound healing process is compromised and can lead to a greater amount of scar tissue and regenerated tissue that has only a percentage of the original tissue’s functional activity (139, 140).

Depending on the extent of separation of the edges of the wound, healing can begin from the sides inward, or from the base upward. During the first few days of wound repair, epithelial cells and fibroblasts migrate across the wound surface into the regenerating tissue where they proliferate and differentiate. Such cells are specialized for the synthesis and secretion of the ECM substances and are fundamental to the architectural repair of the tissue framework. Fibroblasts are known to be very versatile cells that can reversibly transform into highly differentiated cells required for the connective tissue within which they are found. Differentiated cells secrete the types of collagen and ground substances needed for the repair and regeneration of that tissue.

## Inflammation Can Induce Cancerous Growth

Physiological inflammation that occurs during wound healing involves many of the processes associated with *de novo* tumor development as well as mechanisms that endow cancers to metastasize (141–143). The exact directionality of whether inflammation causes carcinogenic processes, or that tumor cells induce local inflammatory responses to facilitate their rapid growth and dissemination is unclear. Indeed, it is likely that there are reciprocal interactions between the host and tumor mediated through inflammatory processes that promote tumor initiation and progression. Overall, the current literature suggests that a lack of resolution to inflammation leads to chronic inflammatory signaling that is intimately linked to promoting the development and progression of cancer.

Tissues involved with cancerous growth have long been known to involve inflammation (111, 141). Mechanistic studies have demonstrated that components of inflammation, such as reactive oxygen species (ROS) and growth factors, can promote both the initiation of neoplastic cells and their proliferation (142, 144). While ROS and proteolytic enzymes produced by neutrophils and macrophages are key contributors to a favorable immune response to the stressed tissue, both superoxide anion and hydrogen peroxide have been shown to induce DNA damage that can contribute to mutagenesis, potentially giving rise to neoplastic cells (145). Unresolved (chronic) inflammation, therefore, would not only prolong immune infiltration at the site of injury, but could promote secondary genetic mutations that could exacerbate malignant conversion of otherwise benign neoplastic cells.

**Figure 4:**
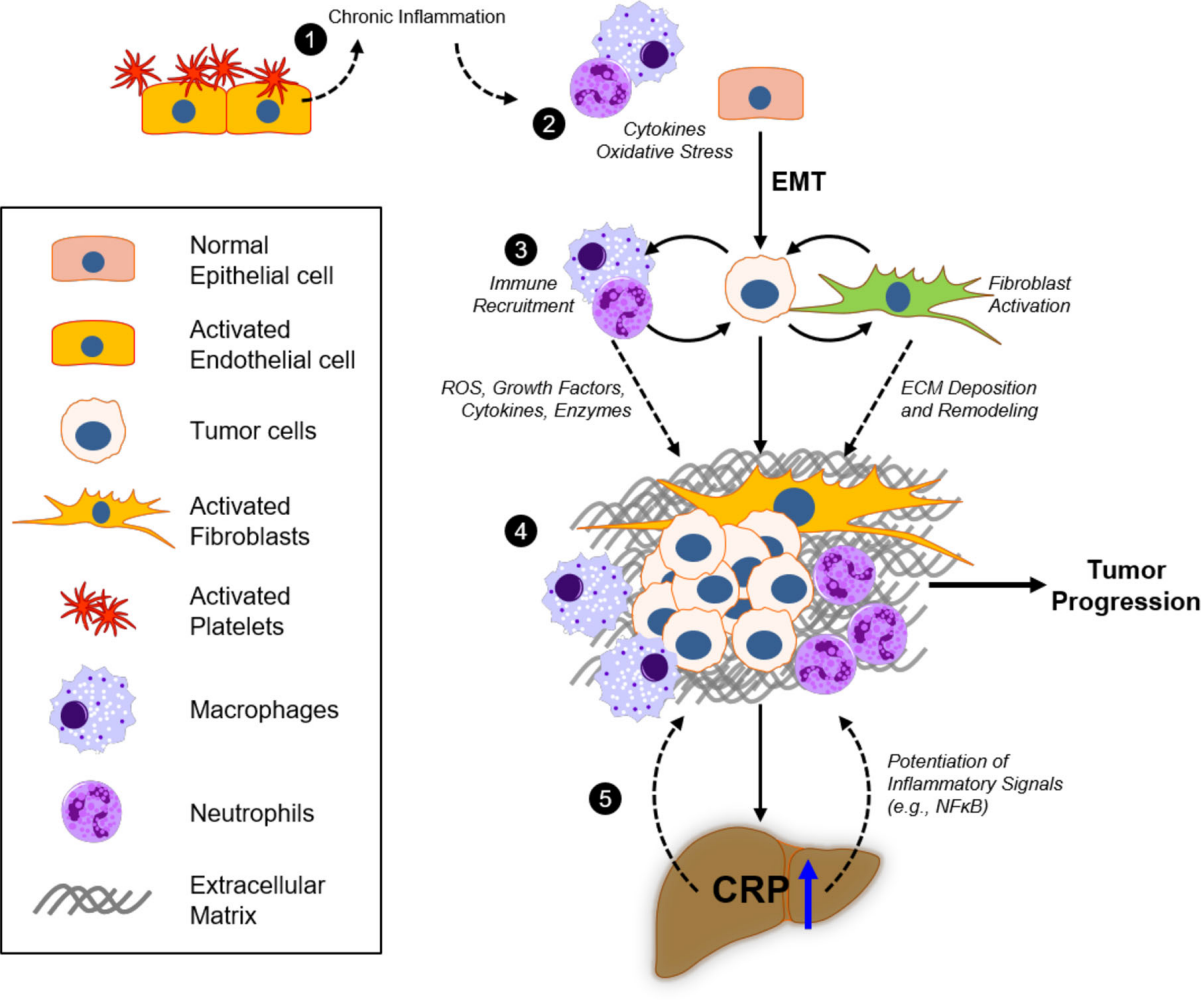
Inflammatory responses of CRP in the extracellular matrix and tumor microenvironment. 1) Platelet recruitment to damaged tissue and fibrin accumulation represent acute phase inflammatory responses that, if injury remains unresolved, will contribute to excessive chronic immunoreactivity. 2) Continuous oxidative stress (reactive oxygen species; ROS) and cytokine production by activated macrophages and neutrophils promote tumorigenicity in epithelial cells, which can promote epithelial-to-mesenchymal transition (EMT) as a result. 3) Deposition of the extracellular matrix (ECM) components, including fibronectin, collagen, laminin, and fibrin, in the tumor microenvironment (TME) by fibroblasts and activated immune cells modulate tumor cell proliferation and invasion. 4) Bidirectional crosstalk in the TME promotes further proliferation of tumor and stromal cells, as well as deposition and remodeling of ECM to promote tumor growth and motility. 5) Excessive cytokine release (e.g., IL-6) from the TME increases systemic circulating levels that promote hepatocyte pCRP production. pCRP secretion and subsequent mCRP-dependent inflammatory signaling (e.g., in involved endothelial cells and neutrophils), as well as its direct action on the ECM, contribute to tumor progression through ROS and cytokine signaling in the TME.

Chronic inflammation could also contribute to pro-tumorigenic processes by increased secretions of growth factors (e.g., PDGF, TGFβ) that promote rapid cell proliferation, as well as cytokines that stimulate cell motility (146). Such inflammatory signals have been observed to induce tumor cell epithelial-to-mesenchymal transition (EMT), a process in which epithelial cancer cells dedifferentiate and adopt a fibroblast-like phenotype to promote rapid growth and enhance pro-metastatic signaling (147, 148), in multiple cancer types. Importantly, this mesenchymal phenotype promotes the production of metalloproteinases to breakdown collagen IV and other ECM proteins to facilitate tumor cell invasion through basement membrane (149) and trans-endothelial migration as cancer cells disseminate into tissues (150, 151). EMT is also known to be associated with upregulated secretion of cytokines and chemokines that allow cancer cells to reprogram surrounding stromal cells to provide a conducive environment for growth and metastasis (152). These data suggest that continuous (chronic) inflammatory responses in any tissue could promote the development of *de novo* malignancies and enhancement of the capacity for tumor cells to metastasize (153). Moreover, inflammatory signaling from the developing tumor could also act systemically to promote acute phase protein production (e.g., hepatic CRP) and provide a positive feedback loop to potentiate this pro-metastatic inflammatory environment (**Figure 4**).

## Cancer Can Induce an Inflammatory Response

Tumors that establish and grow in a tissue environment will induce an inflammatory response that will involve secretion of chemokines that promote immune recruitment (154). Tumor cells will also exploit signaling pathways of localized cells that, under non-cancerous situations would promote fibrosis and tissue repair, in order to foster rapid growth and metastatic potential of the growing tumor mass (149, 155, 156). Under normal physiologic conditions, tissue repair occurs in several phases in response to mechanical injury, infection, or irritation from xenobiotics (156–158). Epithelial or endothelial injury stimulates platelet aggregation and subsequent recruitment of neutrophils and mononuclear cells to the site of injury is then followed by the activation and differentiation of monocytes to polarized macrophages, which secrete growth factors and cytokines to facilitate wound healing through stimulating migration and activation of fibroblasts (159). Activated fibroblasts (myofibroblasts) in turn deposit collagen and remodel the extracellular matrix (160), and in concert with immune cells, promote fibrosis and the resulting formation of granulation tissue to resolve tissue damage.

The tumor microenvironment (TME) has been shown to promote tumor cell proliferation, migration, invasion and intravasation (141, 161, 162) through metabolite and cytokine secretion (163) and production of chemokines involved in immune recruitment (154, 164). Further, the TME favors differentiation of naïve monocytes to M2 macrophages which mediate fibrotic-like responses that facilitate tumor progression (165). Tumor-associated macrophages, in concert with activated fibroblasts and other stromal cell types in the TME, secrete laminin, and collagen to promote tumor cell motility as well as fibronectin, a key modulator of integrin-dependent adhesion and invasion (166–168). As observed in multiple cancers, activated stromal cells in the TME notably induce tumor cell EMT to promote not only proliferation, but also key metastatic processes, such as adhesion, migration, invasion, and colonization (163, 169, 170). Thus, the interplay between tissue structure/function and activated inflammatory responses may contribute to both protection from and exacerbation of disease.

Tumor cells that have undergone EMT secrete a number of growth factors observed during physiologic wound healing, including PDGF (171, 172), TGFβ1 (173), and fibrinogen (174), which are associated with immune cell granulation and enhanced tumor growth. Moreover, mesenchymal-like tumor cells and stromal cells common to the TME have been shown to also secrete cytokines involved in the synthesis of CRP [IL-1β (175)] as well as its secretion [IL-6 (176)], indicating a potential for systemic reaction to the progressing tumor. Locally, this crosstalk by these inflammatory mediators between tumor cells and their microenvironment promotes processes involved in metastasis *in vitro* (163, 169, 177), and markedly enhance the success of tumor growth and metastatic implantation *in vivo* (178, 179). Taken together, these studies indicate that progressing tumors enhance their development and metastasis in part through processes involved in wound healing and pro-inflammatory signaling, suggesting that cancer cell induced inflammation promotes tumor progression and thus disease severity.

The structural support for the regenerating tissue (e.g. basement membranes, non-damaged adjacent tissue) and the connections to the supporting structure tissues are important factors in the effective repair of wounded tissue. Cells growing into the matrix both influence and are influenced by the macromolecules found in the tissue. This influence is mediated within a cell through the intracellular cytoskeleton, composed primarily of actin, intermediate filament, and microtubule proteins. The cytoskeleton helps orient and organize cells within the tissue matrix for optimized function. Connections between the intracellular spaces and the extracellular matrix provide for dynamic and active interactions. Such connections must be reestablished as part of the wound healing process as cells migrate into a wound to regenerate functional, healthy tissues (136, 180–185).

## Proposed Significance of CRP as a Biomarker and as a Biological Response Modifier in Cancer Disease

The clinical studies assessing CRP levels in cancer reviewed above, in light of preclinical data regarding the molecular activity of CRP and its distinctive isoforms in the inflammatory microenvironment, may provide invaluable insight into the contribution of CRP to disease progression of cancers. Moreover, the unique biological activity of mCRP or pCRP could help elaborate the clinical interpretations of CRP levels in patients suffering from cancer, thereby presenting CRP as a potential non-invasive technique to assess the severity of tumor development or progression. In the TME the potential exists for CRP to resolve an inflammatory environment through stimulating retention of monocytes by direct binding to fibronectin (117), which could aid in resolution of a pro-inflammatory (pro-tumorigenic) signaling milieu. Similarly, its ability to limit neutrophil chemotaxis through inhibition of IL-8-dependent migration (121) may also prevent an exacerbation of an inflammatory TME conducive for tumor growth. Conversely, a number of studies suggest the possibility for CRP to positively stimulate leukocyte production of cytokines such as IL-8 and MCP-1 [reviewed in (119)]. Depending on the broader context of stromal cells within the TME, this could indicate that CRP is either 1) enhancing cytotoxic T lymphocyte recruitment and subsequent tumor cell lysis, or 2) prolonging immune recruitment which could potentially lead to a sustained pro-inflammatory, and thus pro-tumorigenic, microenvironment. Therefore, while CRP may facilitate leukocyte retention and subsequent tumor cell lysis early in tumor development, an unresolved neoplastic growth may result in persistent signaling that potentiates both hepatic CRP production and excessive local inflammation, similar to what is observed in other pathologies that fail to resolve following injury and in line with the putative role of CRP in inflammation. While there is tremendous overlap in the matrix characteristics and signaling processes observed in both inflamed tissues and developing tumors, more direct measurement of the physiologic activity of CRP in the TME is both warranted and necessary. Assessing the capacity for CRP to regulate immune cell phenotypes, as well as its ability to modulate behavior of other stromal cells and tumor cells in the TME, can only be accomplished using *in vivo* studies or sophisticated 3D organotypic models (e.g., organoid cultures) to recapitulate the specific TME. Through these methods it may be possible to fully dissect the impact of CRP on the interactions between tumor and stromal compartments in order to assess its role in tumor development and metastasis.

**Figure 5:**
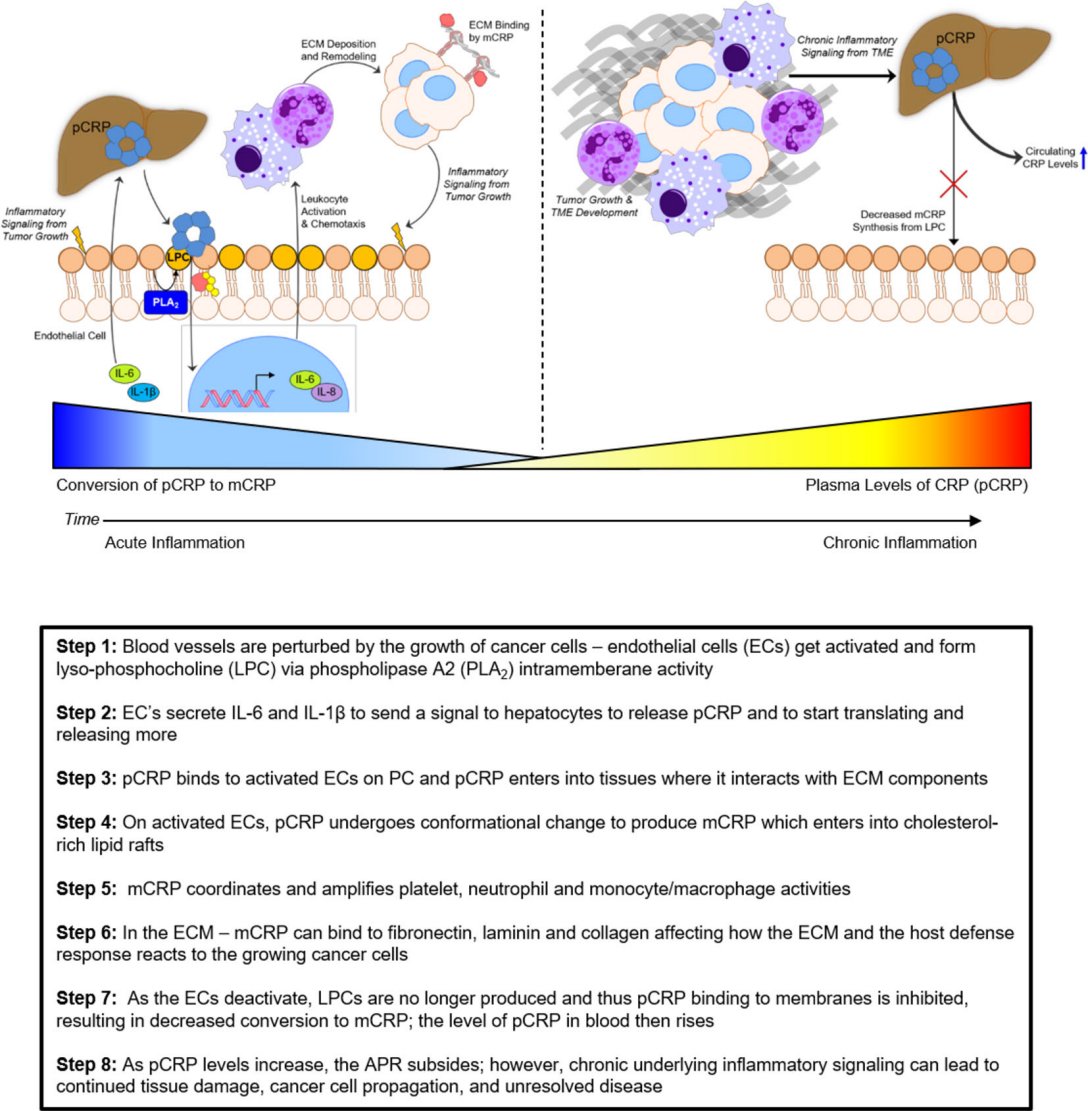
Schematic relationship between pCRP and mCRP as a function of inflammation in cancer.

The emerging relevance of the functional states of CRP isoforms suggest a complex relationship between its response in early inflammation related to *de novo* tumorigenesis and more advanced disease. The activity of mCRP in acute phase response illustrates its tremendous overlap in requisite components and signaling mechanisms of an actively developing tumor milieu. Further, given the observations that systemic levels of both IL-6 and IL-1β are elevated in multiple advanced cancers (186, 187), it is conceivable that the evolving tumor and its microenvironment may contribute to an exacerbation of CRP *de novo* synthesis and continuous secretion, potentially in excess of a slowing rate of conversion to mCRP that results in a demonstrable (and quantifiably significant) increase in systemic pCRP levels (**Figure 5**). This rise in pCRP, and indeed what has been measured in traditional clinical assessments, is thus more likely representative of continued tissue damage resulting from persistent development of neoplasms *in situ*. However, whether the rate of conversion of pCRP to mCRP during tumorigenesis or metastatic progression is like that observed in other inflammatory diseases remains unknown and requires exhaustive investigation. These potential relationships merit further preclinical assessment of the activity of mCRP in the growing tumor microenvironment and early metastatic niches of cancers *in vivo* to inform the exact nature of this inflammatory mediator and the significance of pCRP plasma levels throughout disease progression. Moreover, the recent advances in quantifying mCRP through enzyme-linked assays present a potential way forward for not only identifying the significance of mCRP as a diagnostic marker during disease progression *per se* (188), but could also be adapted to evaluate the molecular role of mCRP in cell-cell communication in the TME. Importantly, further development of such assays that can reliably distinguish between mCRP, pCRP, or possibly membrane-bound CRP will be essential in addressing the limitation of the reviewed clinical studies in that they only measure conventional or hsCRP (**Table 1**), which does not allow for a deeper appreciation of the contextual biological activity of CRP. While the predominant utility of CRP as a biomarker has traditionally been as a non-invasive diagnostic, it may also be useful to measure CRP by immunohistochemical methods or in tumor explant lysates, especially in the evaluation of CRP under controlled conditions in *in vivo* xenograft experiments. In combination with molecular techniques to directly identify interactions of mCRP with tumor and stromal cells, as well as other components of the TME (e.g., ECM proteins), these methods provide approaches that may elucidate the exact impact of mCRP on tumor cell proliferation, migration, invasion, and chemoresistance.

Taking this diversity in the physiologic activity of CRP isoforms into account in the context of cancer may give further insight into the relationship between inflammation and cancer and, moreover, improve the clinical evaluation of cancer progression using this biomarker in patient assessment. Regardless, there are already several clinically important interpretations from the current preclinical and clinical data that may help refine assessment of CRP as a diagnostic tool in cancer, which are presented in **Table 3**. Importantly, the data suggest that pCRP levels exceeding 50–100µg/ml indicates pervasive tissue damage and is associated with poor survival. In general, this may relate to the correlation of high CRP levels and metastatic potential of many tumor types, as outlined in **Table 1**, and is in line with the concept that unresolved inflammation may drive tumor development as well as enhance dissemination and metastasis. This proposed utility of CRP levels to estimate cancer progression are in line with what has been described in assessing disease severity in a number of inflammatory diseases, including analysis of the recent SARS-CoV-2 viral infection (COVID-19) (189). Further evaluation of the role of CRP in cancer will undoubtably improve its ability as a biomarker to indicate disease severity and progression more precisely, and thus may reveal it as an indispensable asset in clinical decision making.

**Table 3:** Proposed diagnostic significance of CRP as a marker of inflammation associated with tissue damage.

1.	CRP blood levels (which only measure the soluble pentameric isoform) should be interpreted as a diagnostic index of tissue health and homeostasis rather than inflammation.
2.	Baseline levels of CRP in health, in controlled disease or in disease remission will be < 10 μg/ml. Levels closer to 1–3 μg/ml are better indicators of good health and control of disease.
3.	As tissues become involved with rapidly growing cancers, hepatic production and release of pCRP increase proportionate to the level by which tissues are affected/damaged by the growing tumors.
4.	Even though stores of CRP are immediately released, there is a lag in quantifying pCRP in blood as it undergoes conformational rearrangement and enters membrane lipid rafts and activates potent pro-inflammatory signaling pathways.
5.	Conformationally-altered CRP is rapidly consumed (proteolyzed); peptides released regulate biofeedback to down-regulate the acute inflammatory response.
6.	When the rate of conformational rearrangement slows, pCRP levels measured in blood increase.
7.	If tissues remain damaged by unresolved disease, blood levels of pCRP will remain elevated.
8.	Elevated pCRP blood levels indicate that a weakened inflammatory response persists which may be insufficient to remove cancerous cells and restore tissue homeostasis.
9.	pCRP levels above 10 μg/ml climbing above 50–100 μg/ml are an index of the degree of ongoing tissue damage.
10.	pCRP levels may also indicate complicating pathologies (e.g. infections).
11.	pCRP values above 100 μg/ml indicate extensive ongoing tissue damage and are consistent with poor prognosis for treatment response and survival.
12.	pCRP levels should be drawn at various times (weekly to monthly) to initially diagnose the presence and severity of primary disease, to assess response to treatment (over time), and to evaluate disease recurrence and prognosis.
13.	pCRP levels taken before and after surgical intervention may help diagnose the response to surgery and the reestablishment of tissue homeostasis.
14.	The significance of hsCRP levels (i.e. CRP levels < 10 μg/ml) is currently unknown.

## Data Availability Statement

The original contributions presented in the study are included in the article/supplementary material. Further inquiries can be directed to the corresponding authors.

## Author Contributions

All authors contributed to the article and approved the submitted version. IR and LP were responsible for the curation of the meta-analysis performed in the review of published works on CRP levels among different cancers.

## Conflict of Interest

The authors declare that the research was conducted in the absence of any commercial or financial relationships that could be construed as a potential conflict of interest.
